# Migration-Enhanced Synthesis of 2D Copper Telluride Ultrathin Thermoelectrics

**DOI:** 10.1021/acsnano.5c21986

**Published:** 2026-04-06

**Authors:** Yu-Chi Yao, You-Chen Lin, Song-Fu Yao, Zhi-Long Yen, Jian-Jhang Lee, Hao-Ting Chin, Ding-Rui Chen, Po-Han Lin, Chia-Chun Chen, Mario Hofmann, Ya-Ping Hsieh

**Affiliations:** † 34879 Institute of Atomic and Molecular Sciences, Academia Sinica, Taipei 10617, Taiwan; ‡ Department of Physics, 33561 National Taiwan University, Taipei 10617, Taiwan; § Nano Science and Technology Program, Taiwan International Graduate Program, Academia Sinica, Taipei 10617, Taiwan; ∥ Department of Chemistry, National Taiwan Normal University, Taipei 11677, Taiwan; ⊥ International Graduate Program of Molecular Science and Technology, National Taiwan University, Taipei 10617, Taiwan; # Molecular Science and Technology Program, Taiwan International Graduate Program, Academia Sinica, Taipei 10617, Taiwan; ∇ Department of Electronic Engineering, 34900 Chung Yuan Christian University, Taoyuan 320, Taiwan

**Keywords:** 2D materials, copper chalcogenides, migration enhancement, van der Waals epitaxy, thermoelectrics, strain sensors

## Abstract

Ultrathin thermoelectric materials offer significant potential for high-performance cooling, on-chip thermal management, and wearable energy-harvesting applications. Copper telluride is a particularly attractive ultrathin thermoelectric owing to its combination of high electronic conductivity and intrinsically low thermal conductivity arising from liquid-like phonon behavior. Realizing these advantages, however, requires the synthesis of highly crystalline Cu_2_Te films which has remained a challenge due to its complex phase diagram and the tendency to form a 3D morphology under high-temperature growth. Here, we report a migration-enhanced chemical vapor deposition strategy that overcomes these limitations and enables the synthesis of ultrathin Cu_2_Te crystals with large grain size and controlled 2D morphology. Introducing a graphene barrier to separate thecopper and tellurium precursors was shown to produce a diffusion-rate-limited growth process that yields ultrathinCu_2_Te crystals with large lateral size. The importance of the copper–graphene interaction was shown through ab initio modeling of the copper transport and experimental observation of ultralow growth temperatures and epitaxial ordering. The resulting 2D Cu_2_Te exhibits superior thermoelectric performance that was exploited in wearable and self-powered sensors. This work establishes a general approach for tailoring surface-migration kinetics in 2D material growth and enables the development of high-efficiency ultrathin thermoelectric devices.

## Graphical abstract

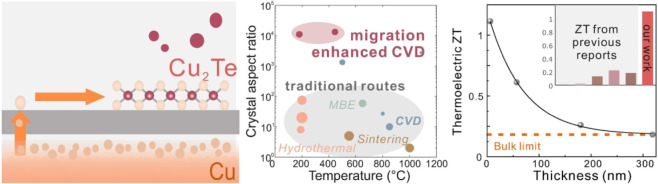

## Introduction

Ultrathin thermoelectrics have received recent attention because of their combination of attractive properties: from a fundamental standpoint, the enhanced scattering at the interfaces decreases thermal conductivity^1^ while quantum confinement of carriers enhances the Seebeck coefficient,^2^ resulting in enhanced performance. Particularly, the discovery of two-dimensional materials has shown the impact of ultrathin dimensionality in overcoming the limitations of the Wiedemann–Franz law.^3,4^

Beyond their fundamental interest, ultrathin thermoelectrics offer practical advantages arising from their anisotropic morphology. Their geometry enables emerging applications, such as planar cooling of integrated circuits^5^ and energy harvesting in flexible and wearable devices.^6^ However, the small cross-sectional area of thin films demands exceptionally high electrical conductivity and low thermal conductivity, imposing stricter material requirements than those in bulk thermoelectrics.

Among potential candidate materials, copper telluride-based 2D materials stand out as promising thin-film thermoelectrics due to their “phonon-liquid electron-crystal” (PLEC) behavior. The strong vibrational anharmonicity of the disordered Cu sublattice promotes multiphonon scattering, which suppresses the lattice thermal conductivity while maintaining good electrical transport.^7,8^

Realizing these advantages, however, requires the synthesis of 2D copper tellurides with high crystallinity and large lateral dimensions, as structural defects and grain boundaries introduce carrier scattering and degrade the thermoelectric performance. Due to several challenges in their synthesis process, 2D copper tellurides have been restricted to small nanoflakes and disordered thin films.^9,10^ First, the complex phase diagram of copper telluride^9^ and the weak Te bonding causes challenges in achieving phase purity and stoichiometry.^11^ Second, the high surface energy of copper telluride favors three-dimensional morphologies at relevant growth temperatures.^12^

Here, we report a migration-enhanced CVD strategy that enables the synthesis of ultrathin Cu_2_Te crystals with an unprecedented aspect ratio. This advance is due to the increased contribution of transport pathways to provide reactants with more time for migration toward favorable lattice sites before bonding. Migration enhancement has shown improvements in crystal quality in diverse material systems, such as diamond,^13^ WS_2_,^14^ and InN.^15^

Unlike previous approaches, which rely on a large spatial separation of the reaction and growth processes, we control the migration of reactants through interaction with a barrier. Exploiting the defect-assisted translocation through a graphene barrier, we achieve selective migration enhancement of copper compared with conventional copper telluride growth. Combined theoretical modeling and experimental characterization confirm the multistep growth mechanism and provide a route toward ultrathin copper telluride at ultralow process temperatures. Moreover, migration enhancement enables the epitaxial ordering of copper telluride on graphene, resulting in large-scale 2D crystals with uniform phase. The resulting high-quality 2D Cu_2_Te exhibits record-breaking thermoelectric performance brought about by ultralow thermal conductivity and a high Seebeck coefficient. We apply this advance to self-powered strain sensors that are promising for use in wearable sensors. Our results demonstrate the potential of 2D material barriers for migration-enhanced synthesis of novel materials for future electronics and energy generation applications.

## Results and Discussions

Copper telluride growth is constrained by the intrinsically low reactivity of tellurium, which usually requires high process temperatures that favor 3D morphologies and are incompatible with the formation of ultrathin films.^11^ To lower the growth temperature without sacrificing precursor reactivity, we spatially separate the Te activation process from the growth process through a two-zone method. In the first zone, Te is heated to 500 °C, and in a second zone, located 30 cm downstream, the growth reaction takes place.

To illustrate the limitations of conventional growth, we react gaseous tellurium with copper foil, following previous work.^16^ In this process, diffusion of Te into the Cu crystal leads to topotactic conversion into copper telluride (Figure 1a). Despite the use of planar copper surfaces, scanning electron microscopy demonstrates complex three-dimensional structures instead of planar films (Figure 1b). This morphology originates from the strain buildup during the diffusion of Te into the Cu crystal due to the mismatch in lattice constants.^17^

**Figure 1:**
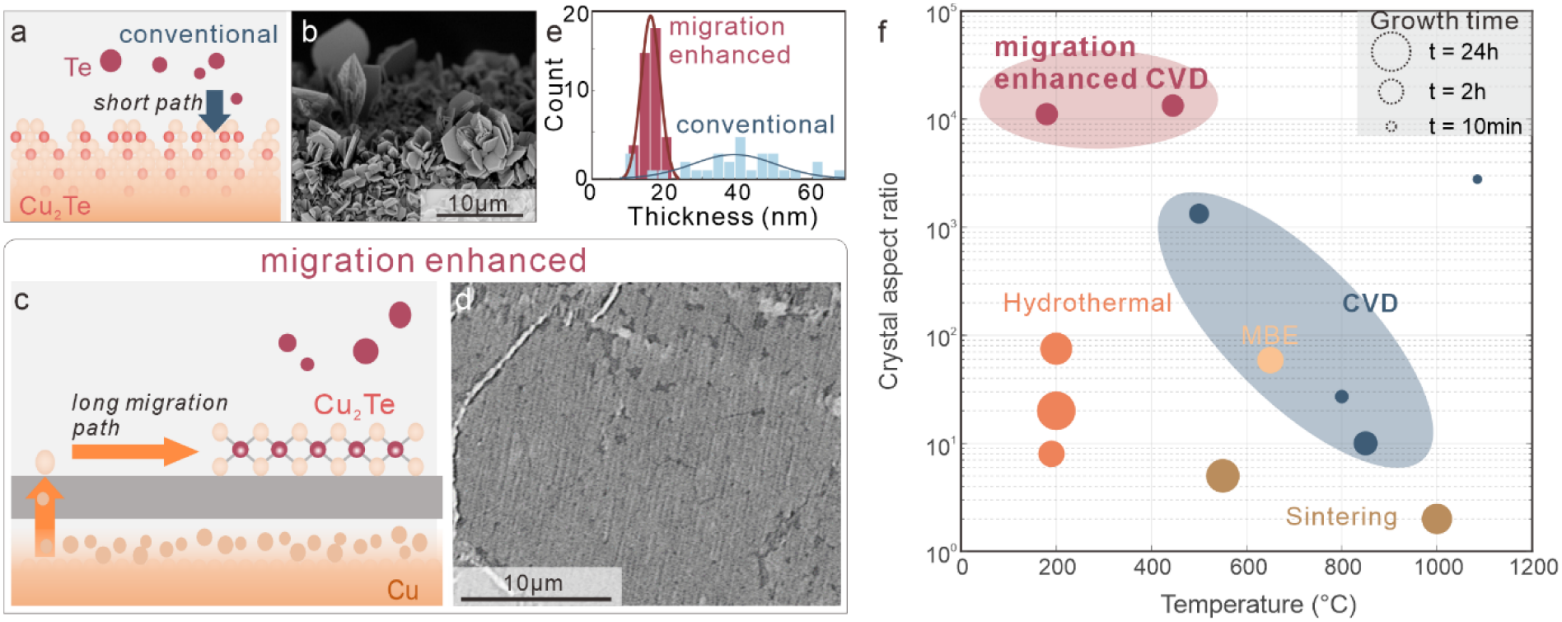
Migration-enhanced copper telluride growth. a) Schematic of reactant migration for conventional tellurization. b) Scanning electron micrograph of copper telluride grown by conventional tellurization. c) Schematic of migration enhancement by a graphene barrier. d) Scanning electron micrograph of resulting thin-film copper telluride. e) Comparative histogram of thicknesses observed for conventional and migration-enhanced CVD. f) Comparison of growth temperature, growth duration, and lateral size/thickness aspect ratio of our growth results to previous reports.^11,20,21^

We transitioned from the conventional to migration-enhanced growth by introducing a layer of graphene on top of the copper substrate prior to growth. Previous work showed that graphene can limit the interaction between the gaseous precursor and the solid precursor, thus increasing the diffusion pathway of reactants^18^ (Figure 1c). The effect of the migration enhancement is apparent from the film-like morphology of the resulting material (Figure 1d).

The observation of a significant change in structure upon the introduction of a graphene layer is quantified by statistical characterization of the thickness (Figure 1e). Compared to conventional tellurization, the migration-enhanced growth shows a 3-fold decrease in average thickness. Despite the significant change in morphology, the stoichiometry of Cu_2_Te was retained, as confirmed by X-ray photoelectron spectroscopy (XPS) (Supplementary Figure S3).

We quantify the observed change in thickness by introducing the parameter of the crystal aspect ratio that represents the ratio of crystal size to crystal thickness. We find that our migration-enhanced growth produces 2D copper tellurides with the highest reported crystal aspect ratio. Moreover, migration-enhanced growth can be achieved at short growth durations and low temperatures (see Methods for detailed growth conditions), resulting in a thermal budget that is among the lowest reported values (Figure 1f). This advance helps to decrease the competition between different phases and limits the temperature-induced formation of the 3D structures. Moreover, the low process temperature could make copper telluride growth compatible with polymeric substrates and soda-lime glass, enabling the application to flexible and low-cost thin-film thermoelectrics.^19^

### Mechanism of the Migration-Enhanced Growth

To understand the mechanism of migration-enhanced growth, we conducted a detailed characterization of the synthesized material. Transmission electron microscopy was employed to establish the cross-sectional structure of the grown film. Previous work on planarized reactions demonstrated the formation of thin Cu_2_S films under the graphene barrier.^18^ In our case, however, Cu_2_Te is grown on top of graphene rather than underneath (Figure 2a). The difference in flux direction compared to that in previous work indicates the inability of Te to permeate the graphene barrier. We conducted ab initio calculations that show a large positive energy barrier to Te translocation through graphene (Figure 2f), confirming our experimental observation (more details in the Supporting Information). Consequently, Cu needs to migrate from under the graphene to the surface to form copper telluride (inset of Figure 2).

**Figure 2:**
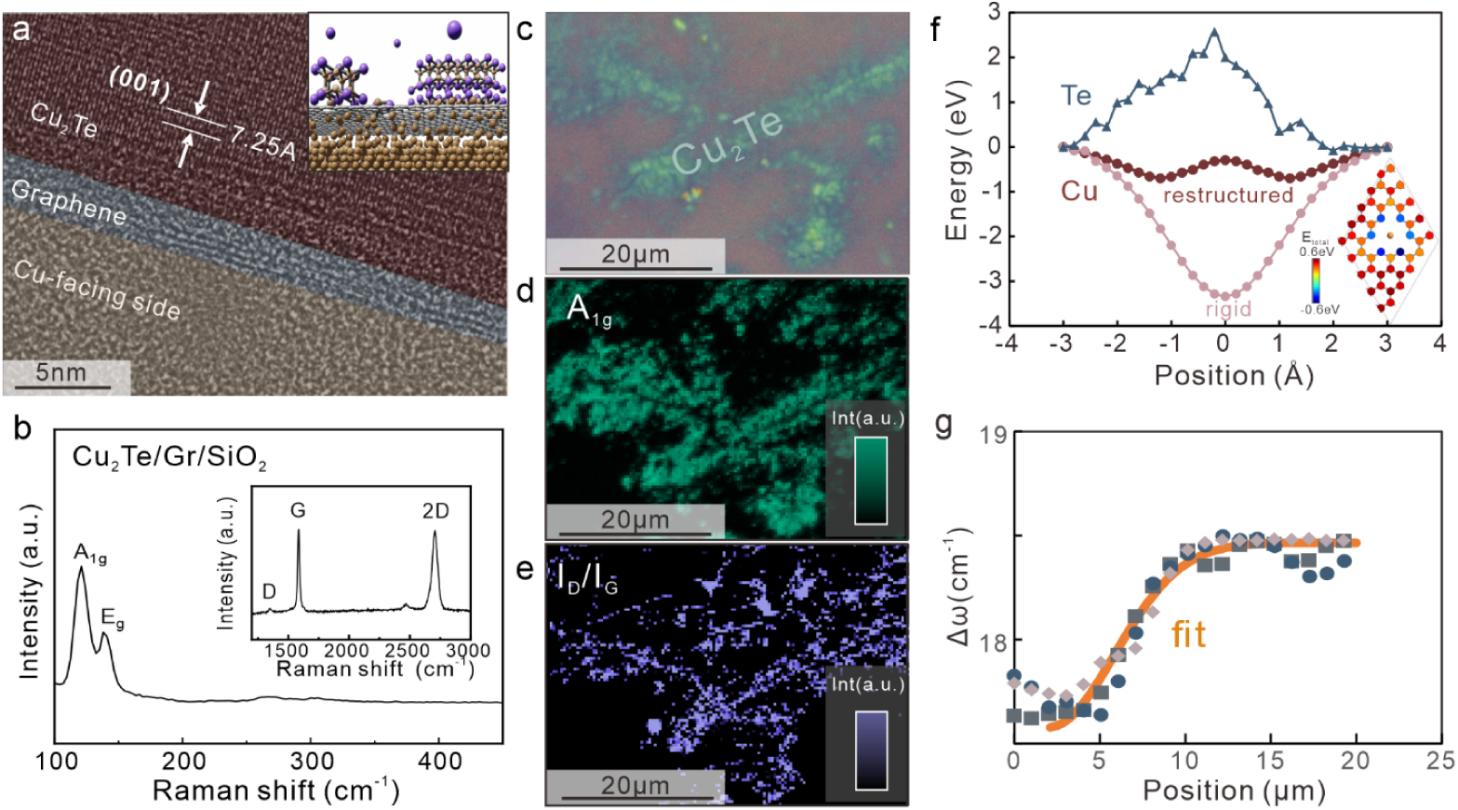
Characterization of the growth process. a) False-color cross-sectional transmission electron micrograph showing Cu_2_Te flake grown on top of a graphene bilayer with an e-beam-induced amorphous transition region.^18,23^ b) Representative Raman spectra showing Cu_2_Te-related low-energy phonon peaks; (inset) high-energy Raman region showing graphene-related features. c) Optical micrograph of the Cu_2_Te inhomogeneous growth pattern. d) Collocated spatial map of the Raman A_1g_ feature demonstrating the distinction between grown and empty regions. e) Collocated spatial map of the Raman *I*_D_/*I*_G_ feature showing the correlation in feature location with d). f) Simulated energy-distance diagram for copper and tellurium migration through a graphene trimer defect using a rigid (Cu) or reorganizing (Cu, Te) graphene sheet. (Inset) Calculated energy contour of a copper atom 2.18 Å above the graphene sheet showing attractive and repulsive contributions upon reorganization. g) Energy difference between the Raman A_1g_ and E_g_ features as a function of separation from the copper ingress point for three different locations, showing a decrease in thickness with migration path length; the fit assumes a relation of: Δ*ω* ∼ *ω*_0_ – *αδ*, where *ω*_0_ represents the peak separation for bulk Cu_2_Te.

Raman spectroscopy is a powerful tool for investigating the growth process , as it can establish both the properties of copper telluride and the condition of the graphene barrier. Representative spectra show low-frequency modes that agree with previously reported signatures of Cu_2_Te vibrations such as the out-of-plane breathing mode from chalcogen atoms (A_1g_) at 121 cm^–1^ and in-plane shear motion (E_g_) at 138 cm−1 ^11^ (Figure 2b). Higher-frequency features are characteristic of graphene, such as the D-band and G-band, and confirm the collocation of Cu_2_Te and graphene (inset of Figure 2b).

Spatial maps of the Cu_2_Te A_1g_ feature and the graphene D/G ratio were acquired for a region that shows a nonuniform concentration of Cu_2_Te (Figure 2c,d,e). We see a clear correlation of the A_1g_ intensity with the color contrast, indicating that it is a good measure of the thickness of Cu_2_Te. The A_1g_ feature is higher in regions that show a high D/G ratio. Since the D/G ratio is a measure of the graphene defectiveness, we conclude that Cu_2_Te is growing preferentially in regions of high graphene defectivity, relying on the presence of defects in graphene.

Ab initio modeling was conducted to identify the mechanism of defect-assisted Cu_2_Te growth. DFT simulations of the translocation of a Cu atom through a graphene trimer defect were conducted (details in the Supporting Information). When only the Cu position is optimized with respect to a rigid graphene sheet, an energy barrier of 2.7 eV in height is observed. This value is close to previous calculations for the adsorption of Cu on single graphene defects.^22^ However, more detailed calculations reveal that the carbon atoms are reorganizing to accommodate the Cu atom, which decreases the translocation energy barrier by 80%. Moreover, the energy contour for the copper translocation is significantly altered by this Cu–C interaction: whereas the rigid graphene case shows a simple parabolic energy curve with a minimum in the graphene plane, the reorganization produces a repulsive force that aids in the translocation of Cu through the graphene sheet (Figure 2f). This repulsive potential is also evident in the potential energy curves along the graphene plane (inset of Figure 2).

We provide additional evidence for the proposed defect-assisted permeation process by analyzing the dependence of the Cu_2_Te growth rate on the separation from defects. Optical microscopy reveals that Cu_2_Te growth produces thicker films in regions close to extended graphene defects, such as grain boundaries or nucleation points. This observation is quantified by Raman spectroscopy. The peak distance between the Raman E_g_ and A_1g_ features can be used to estimate the thickness of layered 2D materials due to the sensitivity of the out-of-plane mode to adjacent layers. Indeed, we observe a clear dependence of Cu_2_Te thickness on the distance from the graphene grain boundaries. The similar decrease in thickness with separation for 3 different sample locations indicates a shared mechanism that is described in a later part.

Finally, we confirm the defect-assisted Cu translocation process by controlling the concentration of graphene defects. When the graphene defectiveness is increased through UV-ozone-based oxidation, enhanced Cu_2_Te growth is observed close to the introduced defects, whereas the extent of Cu_2_Te remains unchanged (Supplementary Figure S10).

The observed efficient translocation of Cu is a requirement for diffusion-enhanced growth. For the lateral transport of species along the graphene sheet to become the rate-limiting step of the growth process, all other steps must be comparably faster. If this requirement is fulfilled, the growth rate of Cu_2_Te would be controlled by the flux of copper atoms toward the Cu_2_Te. This flux has a characteristic temporal and spatial dependence that, in the simplest case (large separation between neighboring defects, constant copper atom supply), follows a 1D diffusive model,

1 $$J_{\mathrm{Cu}}\left(x\right)=D_{\mathrm{Cu}}\frac{d\left[c_{\mathrm{Cu}}\right]}{dx}=\frac{c_{\mathrm{Cu}0}}{\sqrt{\pi D_{\mathrm{Cu}}t}}\exp\left(-\frac{x^{2}}{4D_{\mathrm{Cu}}t}\right)$$

where *c*_Cu0_ is the concentration of copper at the location of the graphene defect serving as the copper ingress point, *D*_Cu_ is the copper diffusion coefficient, *x* is the separation from the defect, and *t* is the growth time.

We find that the Cu_2_Te thickness measured by Raman spectroscopy across the sample fits the prediction of the spatially varying copper flux very well (Figure 2g). From fits to the model, a diffusion coefficient of 1 × 10^–7^ cm^2^/s is extracted, which is close to reported surface diffusion coefficients of copper on metals.

Moreover, we conduct AFM-based characterization of the Cu_2_Te single-crystal thickness vs growth time and observe a square root dependence that also agrees with our model (Supplementary Figure S7).

Our results demonstrate that the rate-limiting step in the growth of Cu_2_Te is the transport of Cu from the graphene translocation point to the growth region, providing evidence of migration-enhanced growth.

Since the other process steps must be faster than the diffusion process, their activation energy must be below 0.9 eV. This estimate agrees with our simulation for copper translocation and also helps us establish the origin of the copper flux:

It may be intuitive to assume that copper is supplied from the surface of the underlying copper crystal. However, our DFT calculations show that the liberation of a Cu atom and simultaneous vacancy formation requires much higher energy than the estimated activation energy (∼4.99 eV) and is not significantly affected by the presence of graphene (more information in the Supporting Information). Instead, previous work emphasized the importance of surface kinks and edge steps as sources of migrating metal atoms.^24^ To investigate this hypothesis experimentally, we correlate the migration-enhanced growth process with the surface texture of Cu. The annealing step prior to graphene growth produces large Cu crystal grains, but due to the low thickness of the Cu foil, exotic textures are stabilized.^25^ When comparing the growth of Cu_2_Te on adjacent Cu(113) and Cu(114) grains, we observe a high concentration of copper telluride formation on the more highly textured surface (Figure 3a,b). This effect is due to the larger number of atomic steps on this surface and the resulting under-coordination of Cu atoms, resulting in a decrease of Cu stability.^26^

**Figure 3:**
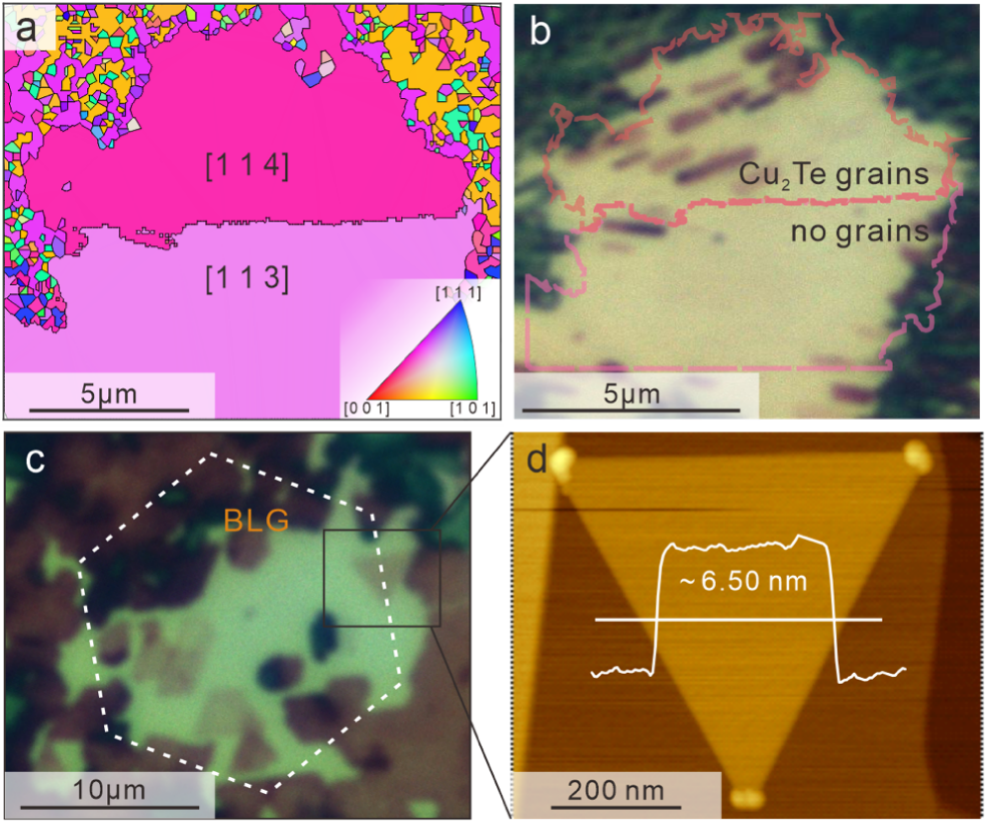
Control of the migration process. a) Electron backscatter diffraction (EBSD) image of two copper grains with different surface orientations. b) Corresponding optical micrograph showing a high concentration of Cu_2_Te grains in the region with [114] texture and no grains in the [113] textured region. c) Optical micrograph of the bilayer region with a low concentration of grains. d) Atomic force micrograph of an individual grain.

In addition to providing a tool for studying the Cu_2_Te synthesis process, migration-enhanced growth helps to enhance the aspect ratio of copper telluride toward ultrathin 2D crystals. Due to the additional activation energy required to overcome the step edge (Ehrlich–Schwoebel barrier), the growth under low-flux conditions is preferentially lateral. Consequently, migration-enhanced growth of 2D materials are preferred at a low flux of copper atoms.

Based on the presented analysis of the growth process, low-flux conditions are reached when the density of effusion points in the graphene plane is reduced. For this purpose, we utilized bilayer graphene regions that occur naturally within CVD-grown graphene single layers. These islands exhibit fewer defects to serve as starting locations for copper migration, thus restricting Cu_2_Te growth. Indeed, we observe a decreasing Cu_2_Te coverage when moving from the edge of the bilayer island into its center (Figure 3c).

By combining low-index copper textures and bilayer graphene, we can produce single-crystal Cu_2_Te flakes with ultralow thickness (Figure 3d). The triangular shape of the flakes is consistent with an edge-attachment growth mechanism for 2D materials^27,28^ and indicates that copper will migrate toward an outgrowing Cu_2_Te crystal and attach at the edge. The increased stability of the zigzag edge will favor this termination, which will subsequently be tellurized.

### Ordering of Cu_2_Te on Graphene

Based on the presented results, graphene has several important contributions to migration-enhanced growth. First, its impermeability to Te emphasizes the migration of Cu as the dominant reaction step. Second, graphene’s high quality yields large regions where Cu cannot penetrate the barrier and increases the migration pathways toward dozens of micrometers. Third, the weak van der Waals interaction of the graphene layer^29^ enhances the efficiency of Cu migration and enables growth at low temperatures.

A final appealing aspect is the large extent of single-crystalline graphene domains that make it suitable as a template for seamlessly merging 2D materials grown in epitaxial registration on it.^30^

We demonstrate the feasibility of this process to realizing large-scale single crystals by investigating the alignment of adjacent Cu_2_Te grains. Electron microscopy indicates a preferential alignment of the grains (Figure 4a) that is confirmed by statistical analysis (Figure 4b) and suggests epitaxial ordering of two 3-fold symmetric systems.^31^

**Figure 4:**
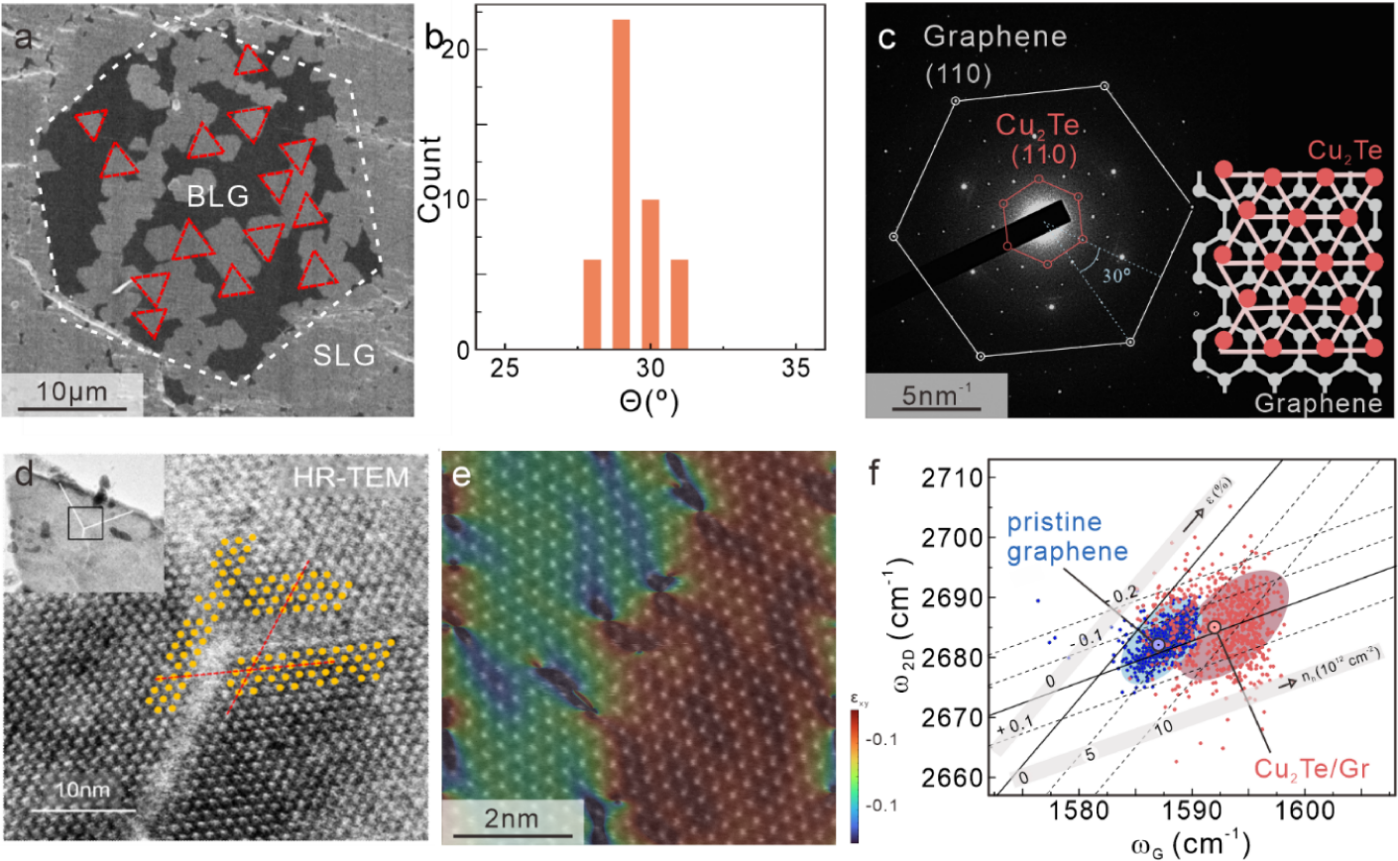
Emergence of Cu_2_Te crystalline ordering through a graphene interaction. a) Scanning electron micrograph of Cu_2_Te grains on bilayer graphene with an indication of their edge orientation. b) Histogram of edge orientations demonstrating preferential alignment. c) Selected area electron diffraction (SAED) pattern of Cu_2_Te on graphene showing high crystallinity of both materials and a relative 30° rotation. (Inset) Depiction of epitaxial alignment between both materials. d) Transmission electron micrograph of interfacial region between merged grains with an indication of alignment. e) Overlay of high-resolution transmission electron micrograph and geometric phase analysis showing perfect crystallographic stitching at the interface between two merged grains that exhibit a rotational distortion. f) Correlation analysis between the Raman 2D-band and Raman G-band showing no significant broadening of the strain distribution of graphene prior to and after Cu_2_Te decoration.

To further corroborate epitaxy, we conducted selective area electron diffraction (SAED) (Figure 4c). We observed two hexagonal patterns that correspond to the predicted lattice structures for Cu_2_Te and graphene.^11,32^ The extracted lattice constants are Cu_2_Te (*a* = 4.30 Å)^11,33^ and graphene (2.46 Å), which are close to reported values. This epitaxial alignment is observed throughout the sample (Supplementary Figure S5) and can be justified by considering the lattice mismatch in a $\sqrt{3}\times \sqrt{3}R30$ structure (inset of Figure 4c).^34^

Utilizing the extracted lattice constants from SAED in (Figure 4c), a lattice mismatch of 0.9% is calculated. This mismatch is much smaller than that of substrates conventionally used for 2D materials growth, such as *c*-cut sapphire (*a* = 4.76 Å; mismatch = 6.5%)^34^ or mica (*a* = 5.31 Å; mismatch = 4.3%),^35^ indicating that graphene can serve as a preferable template for synthesizing highly crystalline copper tellurides. Moreover, the templating effect of graphene can explain the observed phase purity. Statistical SAED analysis (Supplementary Figure S6) and macroscopic XRD (Supplementary Figure S4) analysis both indicate the homogeneous formation of Nowotny-type layered Cu_2_Te despite its energetic unfavorability compared to other phases.^36^ Moreover, temperature-dependent Raman spectroscopy indicates that the phase is stable over a large temperature window from 100 to 500 K, with no phase change or defect formation (Supplementary Figure S11).

The confirmed epitaxial alignment of Cu_2_Te on the graphene substrate permits the coalescence of flakes into a continuous film due to the alignment of neighboring flakes. To illustrate this idea, we investigated the atomic structure of the interfaces between neighboring flakes by transmission electron microscopy (Figure 4d). Through geometric phase analysis,^37^ we observed a location where a portion of the crystal exhibited slight rotational misalignment, suggesting the merging of two grains. Despite the rotational distortion between the grains, the lattices coalesce seamlessly, illustrating the potential of template engineering for single-crystal deposition^38,39^ (Figure 4e).

The high quality of the merged copper telluride film and the absence of interfacial defects were further confirmed by statistical analysis of Raman spectroscopy. Correlation analysis of the Raman 2D-band and G-band allows inference to the conditions of graphene in contact with Cu_2_Te. We observe that the graphene strain distribution does not significantly broaden upon epitaxy of copper telluride, indicating good lattice matching and small variations in strain within the continuous film (Figure 4f).

### Thermoelectric Performance of Ultrathin Cu_2_Te

The ability to produce 2D Cu_2_Te with high crystalline quality at macroscopic length scales allows us to test the hypothesis of its exceptional thermoelectric performance. First, we demonstrate the dominance of the electronic and phononic properties of Cu_2_Te in the as-synthesized graphene/Cu_2_Te heterostructure. Due to the high mobility and high carrier concentration of Cu_2_Te,^12^ it is expected to exceed graphene’s contribution to carrier transport. Indeed, our electrical measurements indicate that the addition of Cu_2_Te enhances the conductivity of graphene 10-fold (Figure 5a).

**Figure 5:**
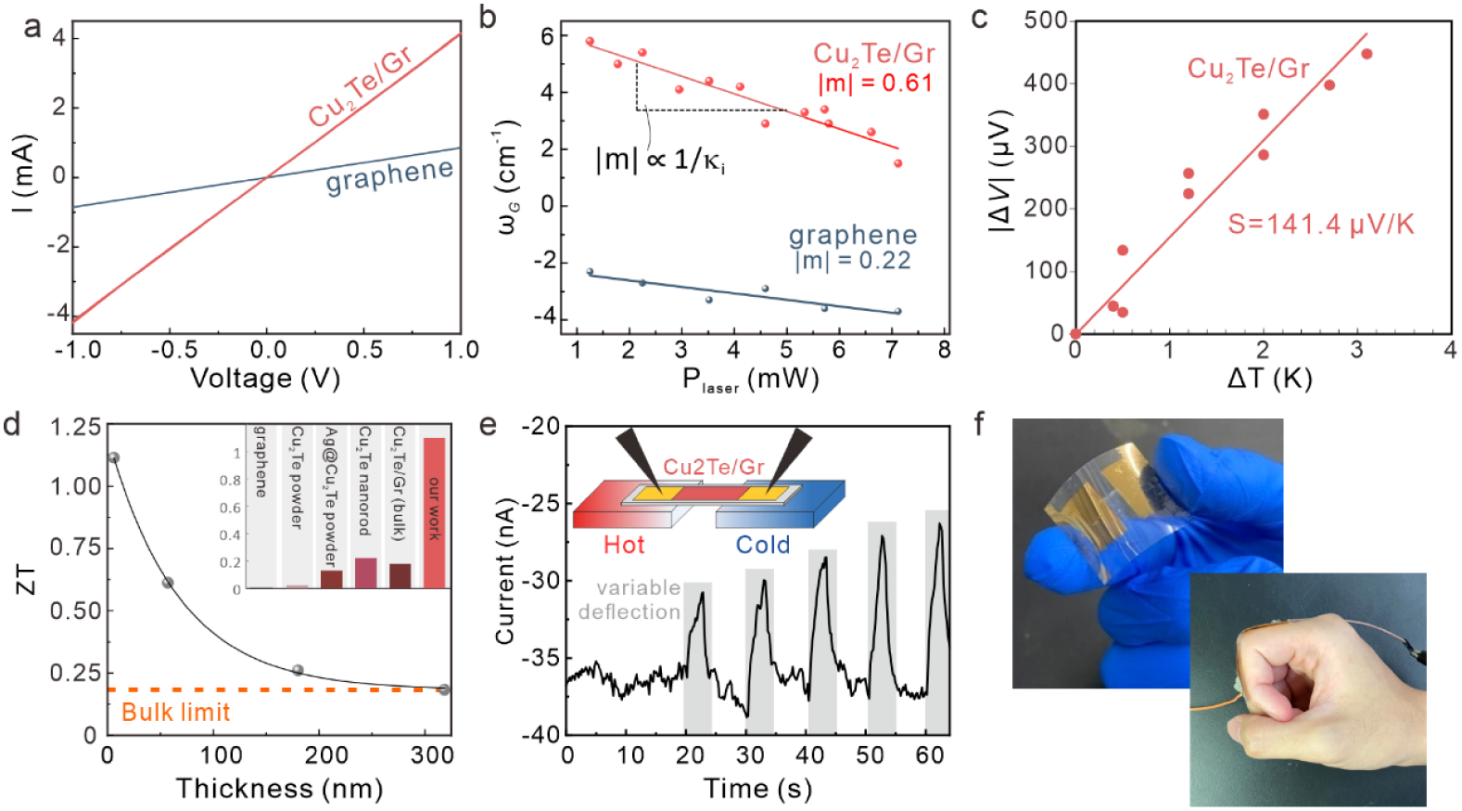
Thermoelectric performance of ultrathin copper telluride. a) Current–voltage characteristics of bare graphene and Cu_2_Te/graphene hybrids, showing significant enhancement in conductance. b) Power-dependent Raman G-band shift for a suspended film showing a decrease in thermal conductivity for the Cu_2_Te/Gr sample compared to pristine graphene. c) Seebeck voltage vs temperature. d) Harman-derived ZT values for samples of different thickness that follow the expected trend for surface-scattering-dominated thermoelectric transport at ultralow thickness. (Inset) Comparison of ZT values in the literature to our work. e) Demonstration of self-powered strain sensor exposed to progressively increasing deformation. (Inset) Schematic of operation. f) Photographs of a self-powered strain sensor and its application to human motion detection.

Second, the liquid-like character of Cu in the Cu_2_Te crystal structure results in low-energy phonons being dominant in the Cu_2_Te crystal.^40^ These phonons cannot easily propagate in graphene due to the low density of acoustic modes,^41^ suggesting that graphene’s contribution to heat conduction is limited. This effect is confirmed by local optical measurements of suspended graphene with and without Cu_2_Te. Through localized heating and characterization of the Raman G-band shift, the thermal conductivity of graphene can be evaluated (more details in the Supporting Information).^42^ While the thermal conductivity of pristine graphene is 822 W/m K, agreeing closely with previous report,^6^ Cu_2_Te lowers the thermal conductivity by 2 orders of magnitude, to 7.42 W/m K (Figure 5b, Supporting Information). This decrease confirms the phonon flux confinement within Cu_2_Te and the suppression of phononic transport.

Finally, we investigate the thermoelectric performance of 2D Cu_2_Te. For this purpose, we extracted the Seebeck coefficient by measuring the voltage at different temperature differences that were calibrated through thermal imaging (Figure 5c and Supplementary Figure S15). A Seebeck coefficient of 141*μ*V/K was extracted which is an order of magnitude larger than bulk Cu_2_Te,^43^ indicating the potential of 2D nanostructuring in enhancing the Cu_2_Te thermoelectric performance.

This enhancement of 2D Cu_2_Te was further proven by measuring the ZT parameter, which is the central figure of merit for thermoelectrics. For thick layers, the Harman-derived ZT value approaches the bulk value,^44–46^ indicating the high quality of the material. However, decreasing the thickness to values comparable to the mean free path of acoustic phonons increases boundary scattering and affects the thermoelectric performance.^47,48^ Indeed, we observe that ultrathin copper telluride layers show superior ZT compared to previous reports and reach unprecedented values of ZT = 1.1 (Figure 5d) (more details on the measurement and good agreement of the value with traditional ZT measurements are provided in the Supporting Information).^44,49^

The high thermoelectric performance of ultrathin Cu_2_Te enables a multitude of novel applications. We demonstrate the operation of a self-powered strain sensor. Upon straining, the resistance of the Cu_2_Te increases, allowing us to extract a high strain gauge factor of GF = 42, which suggests no fracture even at high strain (Supplementary Figure S12). Under a fixed temperature gradient, this strain gauge can produce its own current, which is modulated by the strain-controlled resistance. Consequently, changes in strain can be measured without external voltage (Figure 5e). These appealing properties open up new applications of ultrathin thermoelectrics for self-powered human motion detection (Figure 5f).

## Conclusion

In conclusion, we have demonstrated a migration-enhanced synthesis route that produces copper telluride with high crystalline quality. Through optimization of a graphene migration barrier and copper crystallographic texture, ultrathin 2D Cu_2_Te was produced. The epitaxial alignment of single-crystalline domains enables the formation of high-performance thermoelectrics for future applications in wearable sensors.

## Experimental Section/Methods

### Synthesis of Graphene

The CVD furnace used for synthesizing graphene consists of three heating zones, where a 1 in. quartz tube is used as the reaction chamber. The system is first pumped below 10^–3^ Torr and purged with 10 cm^3^/min of hydrogen. Then, the entire chamber is heated to 1020 °C in 30 min and kept for 70 min to reduce the copper surface. Next, 10 cm^3^/min of CH_4_ is introduced as the carbon source in a mixture of 200 cm^3^/min of H_2_ during the growth process of 6–8 h. Finally, the system is subjected to natural cooling with a constant flow of H_2_ at 10 cm^3^/min.

### Synthesis of Cu_2_Te

The CVD furnace used for tellurization consists of three heating zones, where a 1 in. quartz tube is used as the reaction chamber. 50 mg of tellurium powder (Sigma-Aldrich, 99.8%) is placed in the first zone, while as-grown graphene is located 30 cm away from the solid precursor. Graphite spacers and sheets are placed alternately between Gr/Cu to create a semiclosed but homogeneous atmosphere of reactant vapor. The system is first pumped below 10^–2^ Torr and purged with Ar until it recovers atmospheric pressure. Then, the furnace is heated up to 500 °C (1st zone) and 445 °C (2nd and 3rd zones) for 10 min, maintained for 90 min, and followed by natural cooling at 200 °C. The system is purged with 150 cm^3^/min of Ar during the entire process. For examining the growth behavior, defective graphene is prepared by subjecting the as-grown material to home-built UV ozone cleaner for 30 s while the synthesis temperature of Cu_2_Te on defective graphene is purposely set at 180 °C for less coverage.

### Material Characterizations (AFM, Raman, XPS, XRD, TEM, EBSD)

Thickness and surface potential measurements are conducted with an Atomic Force Microscope from FORCE AFM GENIE E7. Raman spectroscopy was carried out in a home-built confocal micro Raman system at an excitation wavelength of 532 nm. Calibration of spectrum using the Si peak at 520.7 cm^–1^ is conducted prior to every measurement. XPS measurements are carried out using PHI5000 VersaProbe XPS, and all the spectra are calibrated by the C 1s peak (at 284.8 eV) and deconvoluted by the Gaussian/Lorentzian mixture function. Powder diffraction is performed using PANalytical X’Pert Pro X, where *K_α_* emissions of copper are used as the X-ray source accelerated at 45 kV and 40 mA. TEM imaging takes place in JEOL JEM2100F and LVEM 5 SEM/TEM from Delong Instruments, where Cu_2_Te/Gr are suspended on a copper grid (TED PELLA, Prod No. 01883-F) prior to measurements by a wet transfer technique. EBSD characterizations are executed in JEOL JSM-7800F PRIME and are analyzed by OXFORD Nordlys Max3.

For TEM characterization, the copper substrate was removed through a wet-etch step, and the remaining copper telluride and graphene was transferred onto a sacrificial substrate that was ion-milled to expose the cross-sectional plane and retain information on the original orientation.

### Theoretical Calculations

The DFT calculations were carried out using the simulation software QuantumATK (version T-2022.03).^50^ All calculations were performed using a numerical LCAO basis set and the generalized gradient approximation (GGA) with the Perdew–Burke–Ernzerhof (PBE) functional for exchange-correlation interactions.^51^ We constructed Cu slabs with graphene and calculated the vacancy formation energies for different surfaces.

### Device Fabrication and Characterization

Materials are transferred onto a copper grid for TEM measurements using the wet transfer technique. First, PMMA (poly­(methyl methacrylate), Kayaku) dissolved in anisole (Thermo Scientific , 99%) is spin-coated onto the materials as the supporting layer, and then the ensemble is rinsed in 0.5–1 M sodium persulfate solution (Na_2_S_2_O_8_) to etch away the copper underneath. The isolated PMMA/Cu_2_Te/Gr is fished by the silicon substrates or other target materials, and the polymer residues are removed in acetone. A two-terminal device is fabricated using a lithographic patterning system with 50–100 nm Au metallized by thermal evaporation as the contact electrodes. Electrical transport measurements are performed with a Keysight B2912A Source-Measure Unit set up for a multiterminal probing system in ambient conditions.

## Supplementary Material



## Notes

Details of the experimental setup, further characterizations of composition and structural properties of the material, extended simulation results, supporting evidence of the growth mechanism, in-depth discussions of thermoelectric measurements, and comparison of thermoelectric figure of merit (ZT) to previous results (PDF)
