# Supplementary material for: Migration-Enhanced Synthesis of 2D Copper Telluride Ultrathin Thermoelectrics
Source: ACS Nano. 2026 Apr 6;20(15):11765–73. doi: 10.1021/acsnano.5c21986 (PMC13104158; doi:10.1021/acsnano.5c21986)
Supplement: Supplementary file 1 [file nn5c21986_si_001.pdf]

Supporting Information

Migration-enhanced synthesis of 2D Copper telluride ultrathin thermoelectrics

Yu-Chi Yao [a,b ‡], *You-Chen Lin* [a,b,c ‡], *Song-Fu Yao* [a,d ‡], *Zhi-Long Yen* [a,e,f], *Jian-Jhang Lee* [a], *Hao-Ting Chin* [a,e,f], *Ding-Rui Chen* [g], *Po-Han Lin* [a,b], *Chia-Chun Chen* [a,d], *Mario Hofmann* [b], *and Ya-Ping Hsieh* [a *]

[a] *Institute of Atomic and Molecular Sciences, Academia Sinica, Taipei, 10617, Taiwan*

[b] *Department of Physics, National Taiwan University, Taipei, 10617, Taiwan*

[c] *Nano Science and Technology Program, Taiwan International Graduate Program, Academia Sinica, Taipei, 10617, Taiwan*

[d] *Department of Chemistry, National Taiwan Normal University, Taipei, 11677, Taiwan*

[e] *International Graduate Program of Molecular Science and Technology, National Taiwan University, Taipei, 10617, Taiwan*

[f] *Molecular Science and Technology Program, Taiwan International Graduate Program, Academia Sinica, Taipei, 10617, Taiwan*

[g] *Department of Electronic Engineering, Chung Yuan Christian University, Taoyuan, 320, Taiwan*

*Corresponding author Email: yphsieh@gate.sinica.edu.tw

1. Synthesis setup

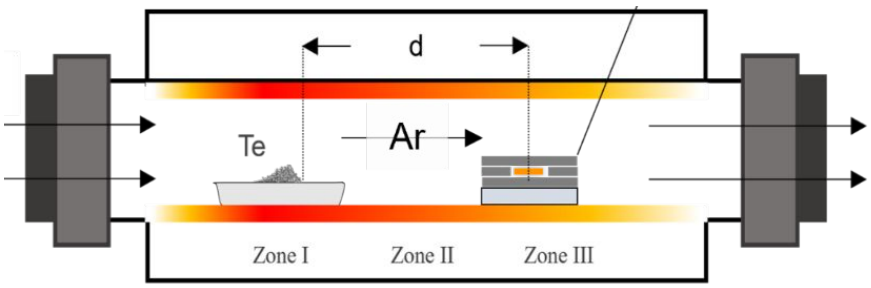

Figure S1 Schematic of copper telluride growth in multi-zone CVD reactor.

2. DFT simulations

To estimate the diffusion behavior of copper with the existence of graphene defect, DFT calculations were performed using QuantumATK (version T-2022.03) with a numerical linear combination of atomic orbitals (LCAO) basis set, and norm-conserving pseudopotentials from the PseudoDojo library were used. The exchange-correlation interactions were treated within the generalized gradient approximation (GGA) using the Perdew-Burke-Ernzerhof (PBE) functional. The Brillouin zone was sampled using a 3x3x1 Monkhorst-Pack k-point mesh, with 1 k-point along the vacuum direction. Also, van der Waals interactions were included using the Grimme DFT-D3 correction. The convergence threshold for self-consistent field (SCF) cycles was set to 0.0027 eV, and the force tolerance for geometry optimization is 0.05 eV·A^{-1}.

Atom translocation through graphene defect

We construct a suspended graphene sheet with 50 carbon atoms. A trimer defect is constructed by deleting 4 neighboring carbon atoms as show in **Figure S2**. A copper atom is placed at the geometry center of the defect and adjusts the out-of-plane (Z direction) distance between copper

atom and the graphene sheet. Two sets of calculations are conducted: 1. single point calculation as a rigid data 2. geometry optimization for in-plane geometry for different distances between copper atom and graphene sheet as a restructured data.

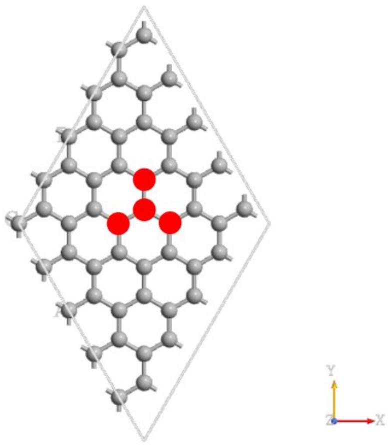

Figure S2 A suspended graphene sheet with trimer defect (red dots)

Calculation of lateral potential energy surface

We place a copper atom in a distance of 2.18A, which represents the stable position of the Cu atom, above the the middle of a graphene trimer defect and calculate the energy difference for copper atom to move in a plane parallel to the graphene sheet.

Copper vacancy formation

We calculate the vacancy formation energy of Cu slabs using DFT calculations.[1] The vacancy formation energy was obtained using the equation:

$$E_{form} = E_{tot}^{defective} - E_{tot}^{surface} + x \cdot E_{tot}^{Cu} \quad (2)$$

where $E_{tot}^{defective}$ is the total energy of a Cu slab with x surface atom removed, $E_{tot}^{surface}$ is the energy of the pristine surface, and E_{tot}^{Cu} is the energy per atom in bulk Cu. Beside vacancy formation energy for Cu, we also place a graphene sheet on the Cu slab to obtain the vacancy formation energy for Cu with graphene. The vacancy formation energy with graphene is 4.99 eV, while it is 4.96 eV without graphene.

3. Photoelectron characterization

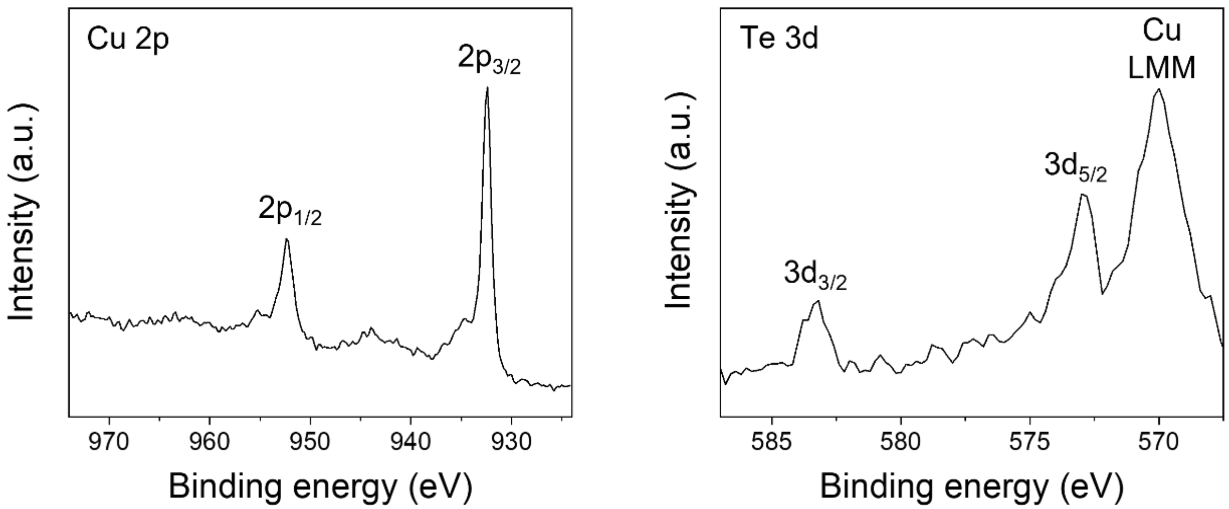

Figure S3 XPS spectra of Cu 2p and Te 3d peaks whose proportion can be converted into a composition of Cu:Te = 2:1

4. Diffraction characterization

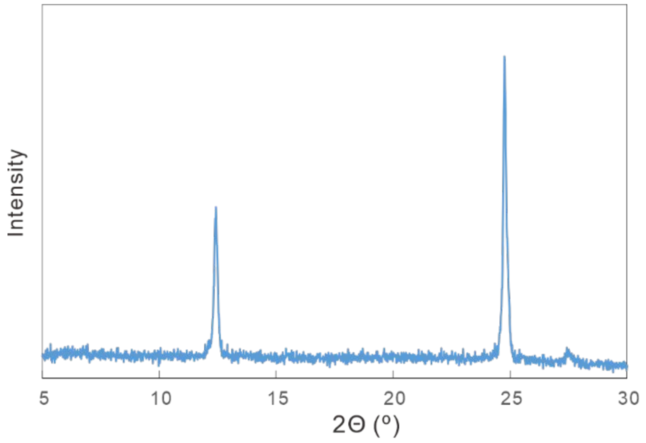

Figure S4 X-Ray diffractogram showing (001) and (002) peak associated with c-axis textured layered material

5. Demonstration of epitaxial uniformity

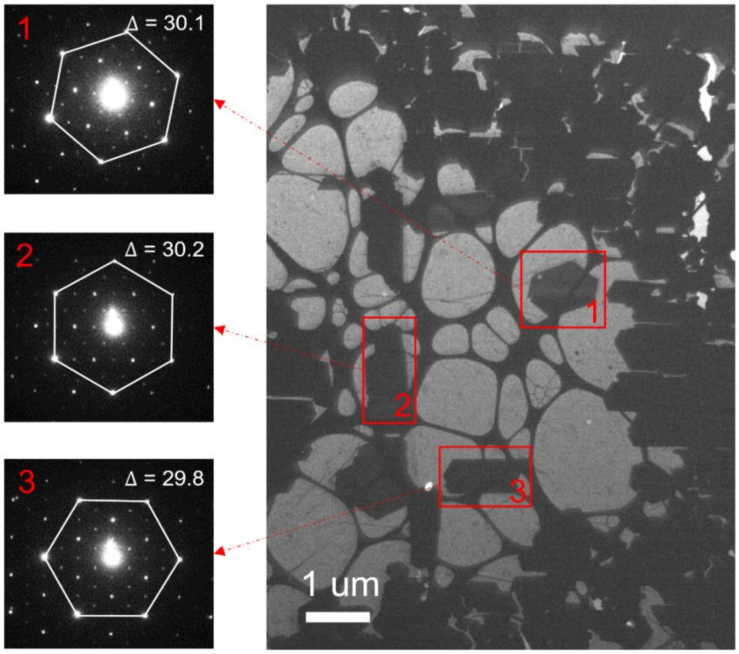

Figure S5 Selected Area Electron Diffraction of different crystalline regions within a TEM grid showing similar alignment between the graphene underlayer and the Cu_2Te grain. The rotational variability of the graphene substrates originates from its polycrystalline nature.

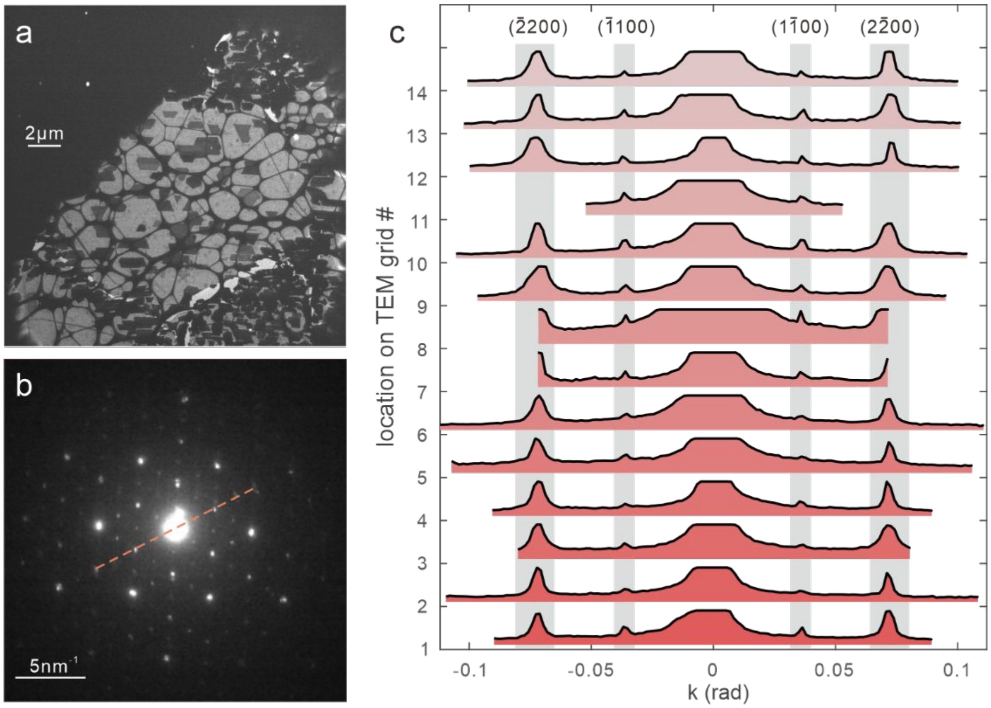

Figure S6 (a) transmission electron micrograph of Cu_2Te crystals within one TEM grid window, (b)representative Selected Area Diffraction (SAED) pattern with indication of the cutting line for intensity measurements, (c) comparison of SAED patterns taken from different TEM grid windows that were several millimeters apart. Analysis of the four characteristic reflexes indicates that the variation in lattice constant across the sample is less than 1%.

6. Growth mechanism studies

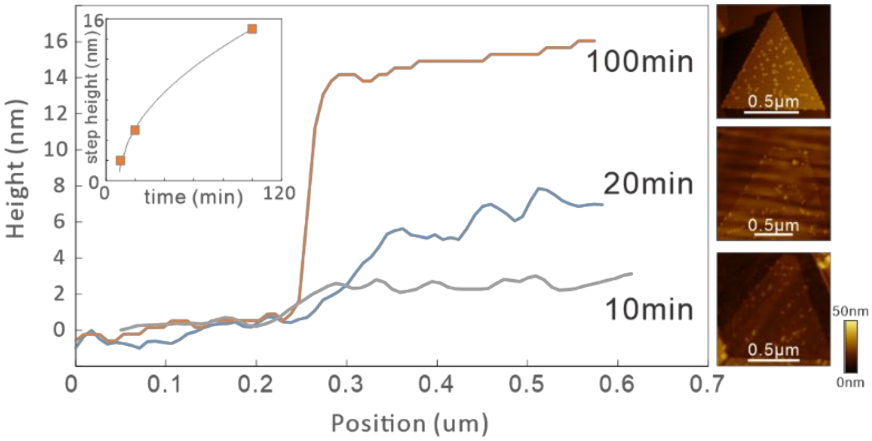

Figure S7 Atomic force micrographs of Cu_2Te grains after different growth durations and their corresponding step profile, (inset) extracted step heights vs growth time with fit to a diffusion-controlled growth mechanism.

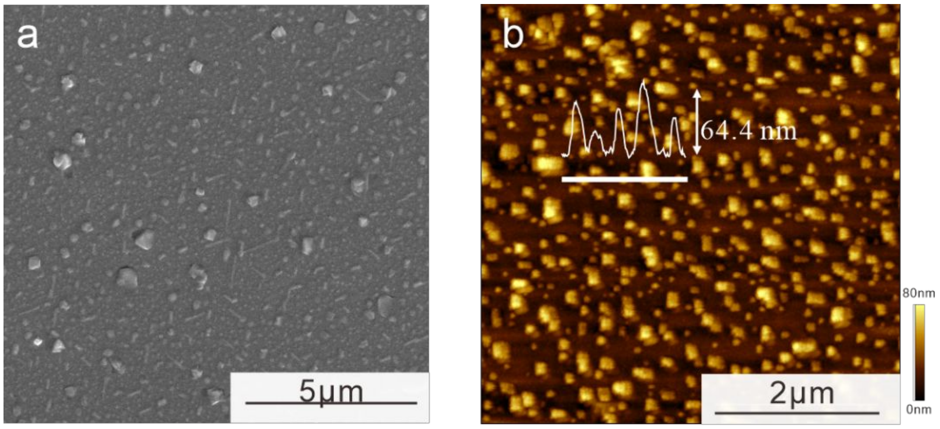

Figure S8 (a) Scanning electron micrograph of growth morphology without graphene layer, (b) corresponding atomic force micrograph confirming growth of thick particles.

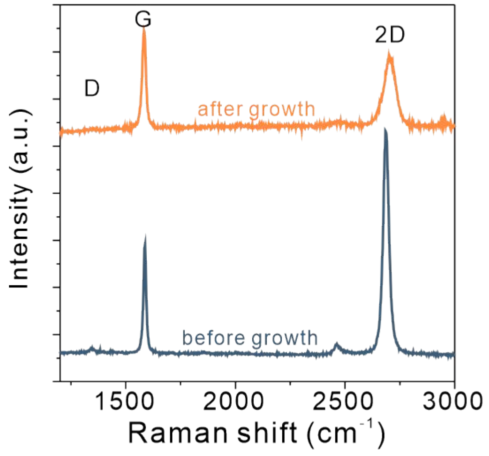

Figure S9 Representative Raman Spectra of graphene before and after Cu_2Te growth showing limited variation in D-Band intensity.

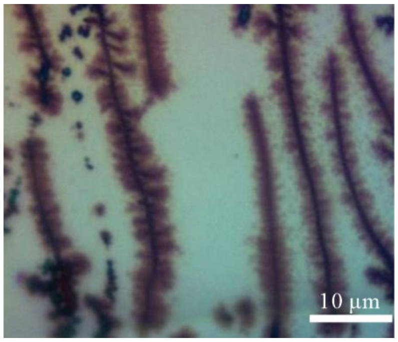

Figure S10 Optical micrograph of growth results on UV-ozone treated graphene showing the formation of line defects and the preferential growth of Cu_2Te around these defect sites. No qualitative change in the morphology of the Cu_2Te was observed that would indicate a change in migration along defected graphene.

7. Phase stability at high temperatures

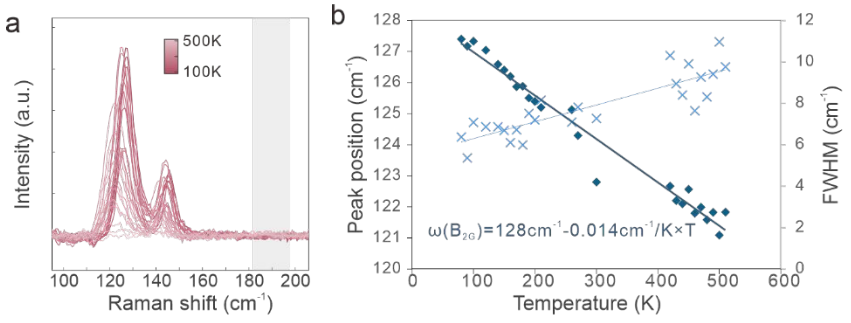

Figure S11 (a) Comparison of Raman spectra obtained at varying temperatures, (b) extracted peak positions and peak widths vs temperature showing a monotonic change without abrupt changes that would indicate phase transitions or defect formation. The fitting line represents a simple Gruneisen model with a parameter comparable to other 2D materials.[2]

8. Strain sensor measurements

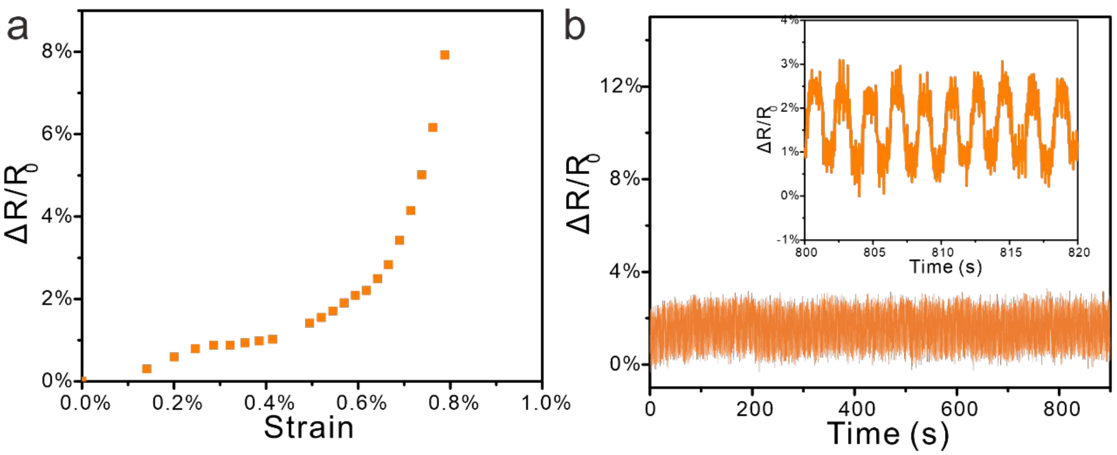

Figure S12 a) Strain-dependent resistance measurements. b) Demonstration of reproducibility after straining for 400 cycles.

9. Thermoelectric measurements

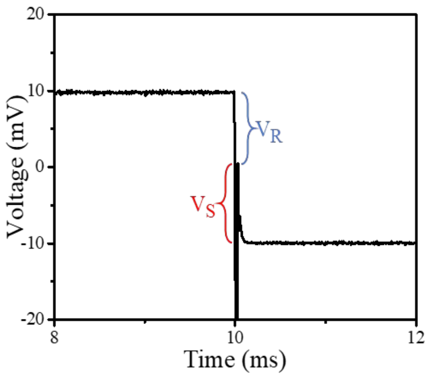

Figure S13 Current-time evolution after heating pulse for Harman ZT measurements.

Contact resistance represent an issue for both electrical and thermal measurements of Cu_2Te due to the mismatch in band structure and phononic structure of contact/substrate and 2D material. To extract the electrical resistivity independent of contact resistance, we conduct TLM measurements where the total resistance was measured as the separation between contacts was varied. The resulting resistivity value of $80\ \Omega \cdot \mu m$ demonstrates the enhanced conductivity due to Cu_2Te.

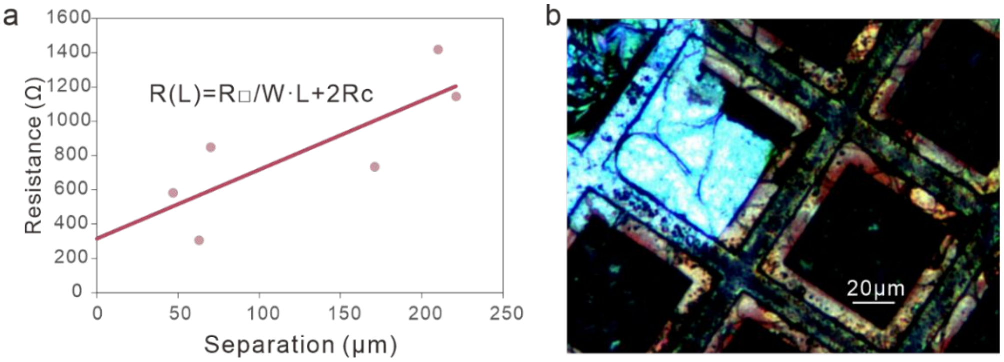

Figure S14 (a) TLM results of separation-dependent resistance showing a linear trend corresponding to materials sheet resistance $R_{\square}$ and contact resistance R_c. (b) Microscope image of suspended Cu_2Te/graphene on TEM grid for Raman optothermal measurements.

The impact of thermal contact resistance can be seen when extracting the Seebeck coefficient. For this purpose, the graphene/Cu_2Te heterostructure was transferred onto a sapphire substrate and contacted by gold electrodes. Two thermoelectric Peltier elements were used as heater and heatsink, respectively, to produce a fixed temperature gradient. Thermal imaging shows a significant thermal contact resistance between the Peltier elements and the sample (**Figure S15(a)**), resulting in a large temperature drop at the contacts (**Figure S15(b)**). The temperature gradient was extracted from the thermal images at the location of the Cu_2Te contacts. The Seebeck coefficient data presented in (**Figure 5(c)**) represents results from several devices, showing the reproducibility of the measurement.

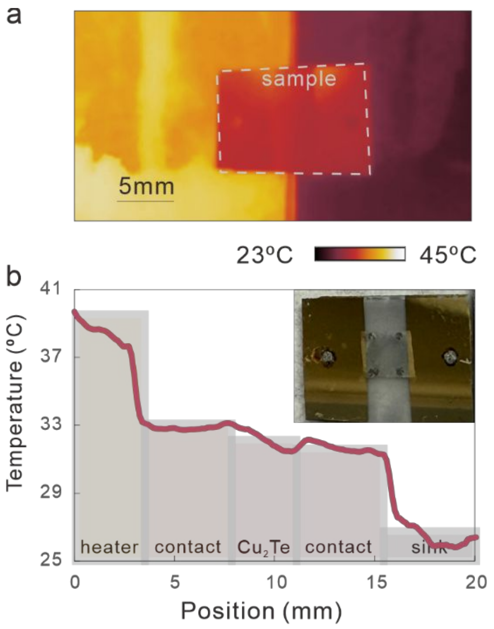

Figure S15 (a) Thermal image of the temperature distribution in the Seebeck voltage measurement system, (b) cross-section of the temperature distribution across the measurement system (inset) photograph of the Cu_2Te device.

The thermal conductivity was extracted from the power-dependent shift of the Raman G-Band in (**Figure. 5(b)**) according to

$$\kappa = \left(\frac{\varkappa_G}{2h\pi}\right) \ln\left(\sqrt{2}\frac{a}{r_0}\right) \left(\frac{\delta\omega}{\delta P}\right)^{-1}$$

, where $\varkappa_G$ is the temperature coefficient of the Raman G-peak shift, h is the thickness of the suspended layer, a is the half-width of the square opening, r_0 is the laser spot size and P is the laser power.[3] Using previous estimates for $\varkappa_G$ to be $-0.016\ \mathrm{cm}^{-1}/\mathrm{K}$ and a thickness obtained by AFM, we arrive at a thermal conductivity of graphene of 822 W/mK which is close to the values reported using the same CVD grown graphene source.[4] The thermal conductivity of the Cu_2Te/graphene

heterojunction, is estimated to be only 7.42 W/mK owing to the larger thickness and higher observed G-band power dispersion. This value is only a rough estimate since it does not account for strain-induced changes to x_G by interaction with Cu_2Te and non-uniform heating. Moreover, the formula assumes perfect thermal contact between the graphene and the substrate acting as a heatsink, which leads to further errors.

To increase the confidence of our thermoelectric measurements we compare the directly measured ZT value by the Harman method to the combination of the individually measured parameters according to:

$$ZT = \frac{S^2T}{\rho\kappa}$$

, where S is the Seebeck coefficient, T is the absolute temperature, κ is the thermal conductivity, and ρ is the electrical resistivity.

Using the experimentally obtained values, we arrive at a ZT value of 1.00 which is comparable to the value obtained by Harman measurements and demonstrates the robustness of the results.

Comparison of thermoelectric performance

ZT	Ref
0.00055	Enhancement of graphene thermoelectric performance through defect engineering[5]

0.022	Thermoelectric properties of Ag-doped Cu2Se and Cu2Te[6]
0.13	Synergistic effect of band convergence and carrier transport on enhancing the thermoelectric performance of Ga doped Cu2Te at medium temperatures[7]
0.22	Fabrication of one-dimensional Cu2Te/Te nanorod composites and their enhanced thermoelectric properties[8]
0.18	Copper telluride with manipulated carrier concentrations for high-performance solid-state thermoelectrics[9]
1.1	our work

Table S1 Literature review of ZT values for related materials systems

(1) Tafreshi, S. S.; Roldan, A.; de Leeuw, N. H. Density Functional Theory Study of the Adsorption of Hydrazine on the Perfect and Defective Copper (100), (110), and (111) Surfaces. J Phys. Chem. C 2014, 118 (45), 26103–26114.

(2) Thripuranthaka, M., Late, D. J. (2014). Temperature dependent phonon shifts in single-layer WS_2. ACS Appl. Mater. & Interfaces, 6(2), 1158-1163.

(3) Balandin, A. A., Ghosh, S., Bao, W., Calizo, I., Teweldebrhan, D., Miao, F., & Lau, C. N. Superior thermal conductivity of single-layer graphene. Nano Lett. 2008, 8(3), 902-907.

(4) Wang, Y. Y., Chen, D. R., Wu, J. K., Wang, T. H., Chuang, C., Huang, S. Y., Hsieh, W. P., Hofmann, M., Chang, Y. H. & Hsieh, Y. P. Two-dimensional mechano-thermoelectric heterojunctions for self-powered strain sensors. Nano Lett. 2021, 21(16), 6990-6997.

(5) Anno, Y.; Imakita, Y.; Takei, K.; Akita, S.; Arie, T. Enhancement of graphene thermoelectric performance through defect engineering. 2D Mater. 2017, 4 (2).

(6) Ballikaya, S.; Chi, H.; Salvador, J. R.; Uher, C. Thermoelectric properties of Ag-doped CuSe and CuTe. J Mater. Chem. A 2013, 1 (40), 12478–12484.

(7) Sarkar, S.; Sarswat, P. K.; Saini, S.; Mele, P.; Free, M. L. Synergistic effect of band convergence and carrier transport on enhancing the thermoelectric performance of Ga doped CuTe at medium temperatures. Sci. Rep. 2019, 9, 8180.

(8) Park, D.; Ju, H.; Oh, T.; Kim, J. Fabrication of one-dimensional Cu_2Te/Te nanorod composites and their enhanced thermoelectric properties. CrystEngComm 2019, 21 (10), 1555–1563.

(9) Ju, H.; Park, D.; Kim, M.; Kim, J. Copper telluride with manipulated carrier concentrations for high-performance solid-state thermoelectrics. J Mater. Sci. Technol. 2022, 129, 190–195.
